# A comparison between drug-eluting stent implantation and drug-coated balloon angioplasty in patients with left main bifurcation in-stent restenotic lesions

**DOI:** 10.1186/s12872-020-01381-9

**Published:** 2020-02-18

**Authors:** Hyungdon Kook, Hyung Joon Joo, Jae Hyoung Park, Soon Jun Hong, Cheol Woong Yu, Do-Sun Lim

**Affiliations:** Department of Cardiology, Cardiovascular Center, Korea University Anam Hospital, 126-1, 5ka, Anam-dong, Sungbuk-ku, Seoul, 136-705 Republic of Korea

**Keywords:** Left main, Bifurcation, Drug-eluting stent, Drug coated balloon, Major adverse cardiac event

## Abstract

**Background:**

The current guidelines recommend both repeat stenting and drug-coated balloons (DCB) for in-stent restenosis (ISR) lesions, if technically feasible. However, real-world clinical data on the interventional strategies in patients with left main bifurcation (LMB)-ISR have not been elucidated.

**Methods:**

Seventy-five patients with LMB-ISR, who underwent percutaneous coronary intervention (PCI) between January 2009 and July 2015, were retrospectively reviewed for the present study (repeat drug eluting stent [DES] implantation [*n* = 51], DCB angioplasty [*n* = 24]).

**Results:**

Analysis of the baseline characteristics showed that the patients in the DCB group had a lower incidence of non-ST segment elevation myocardial infarction/ST segment elevation myocardial infarction at the index PCI (8.3% vs. 25.5%; *p* = 0.12), higher low-density lipoprotein-cholesterol level (92.9 mg/dL vs. 81.7 mg/dL; *p* = 0.09), and more “stent-in-stent” lesions (25% vs. 7.8%; *p* = 0.07) than those in the DES group. A smaller post-procedural minimal target lesion lumen diameter was also noted in the DCB group than in the DES group (2.71 mm vs. 2.85 mm; *p* = 0.03). The cumulative incidence rates of major adverse cardiac events (MACEs) were similar between both groups (median follow-up duration, 868 days; MACE rate, 25% in the DCB group vs. 25.5% in the DES group; *p* = 0.96). The multivariate Cox regression analysis indicated that the true bifurcation of ISR was an independent risk predictor of MACEs (hazard ratio, 4.62; 95% confidence interval, 1.572–13.561; *p* < 0.01).

**Conclusions:**

DES and DCB showed comparable long-term clinical results in patients with LMB-ISR lesions.

## Study highlights

1. Left main bifurcation in-stent restenosis (LMB-ISR) is rare but clinically complex and associated with poor prognosis.
2. Patients with LMB-ISR showed similar clinical prognoses between drug-coating balloon angioplasty and drug-eluting stent implantation.
3. Angiographically, drug-coating balloon angioplasty had a trend of higher binary restenosis and larger late lumen loss compared to drug-eluting stent implantation.
4. True bifurcation in-stent restenotic lesions were the only independent risk predictor for poor clinical outcome.

## Background

Previously, coronary artery bypass graft surgery (CABG) was the standard treatment strategy used in patients with left main coronary artery (LMCA) disease. Cumulative evidence from large observational and randomized clinical trials has suggested that percutaneous coronary intervention (PCI) may be as feasible and safe as CABG in this patient population [1–3]. Thus, the current guidelines recommend both CABG and PCI for LMCA disease depending on its anatomical consideration [4, 5]. However, PCI in patients with LMCA disease has been associated with a higher risk for restenosis and repeat revascularization. In the SYNTAX trial, the 5-year incidence rate for repeat revascularization after PCI for LMCA was 26.7% [6], and in the PRECOMBAT trial, the incidence rate of ischemia-driven target vessel revascularization was 11.4% [7]. More importantly, the lesions and procedural involvement of the left main bifurcation (LMB) have been shown to be a significant predictor of in-stent restenosis (ISR) [8–10]. Stenting at the ostial and proximal segments of the major side branches, including the left anterior descending artery (LAD) and left circumflex artery, could contribute to restenosis in patients who have undergone LMB PCI. Nevertheless, data are scarce on PCI strategies in patients with LMB-ISR.

A previous meta-analysis demonstrated that drug-eluting stent (DES) implantation could be effective in patients with ISR after bare metal stent implantation [11]. However, the effectiveness of repeat DES implantation for ISR after previous DES implantation remains controversial. Recently, drug-coated balloons (DCB) have emerged as a potential alternative for ISR lesions. DCB was reported to be superior to plain balloon angioplasty and non-inferior to repeat DES implantation [12, 13]. However, most randomized controlled studies have excluded patients with left main coronary lesions, and data regarding the ideal PCI strategy for patients with LMB-ISR are still lacking. In this study, we sought to compare the long-term clinical outcomes between DCB angioplasty and repeat DES implantation in patients with LMB-ISR lesions.

## Methods

### Study population

We performed a single-center retrospective study and screened 4258 consecutive patients who underwent PCI between January 2009 and July 2015. Of these patients, 355 had ISR lesions, which were defined as > 50% ISR or within a 5-mm distance from the stent edges according to quantitative coronary angiographic imaging analysis. Overall, 77 patients underwent repeated percutaneous coronary interventions for LMB-ISR lesions. Two patients who had already undergone DCB angioplasty for LMB-ISR lesions were excluded. None of the patients used any other special devices, such as cutting balloons. Altogether, we analyzed data from 75 patients with LMB-ISR lesions who underwent DES implantation or DCB angioplasty.

Clinical data until June 2016 were investigated using medical records and telephone calls. The study protocol was approved by the Institutional Review Board of Korea University Anam Hospital (IRB NO. AN16238–002) and the requirement for written informed consent was waived due to the retrospective study design. The study also complied with the Declaration of Helsinki.

### Interventional procedure

All patients were pre-treated with aspirin (100 mg) and clopidogrel (75 mg) daily. Dual antiplatelet treatment was maintained for at least 12 months in both groups. In addition, interventional procedures were performed according to the standard clinical guidelines. Interventional strategies, including DCB, DES, adjunctive devices, and pharmacotherapy were chosen at the surgeon’s discretion. Balloon predilatation was performed in all patients with ISR lesions. When kissing balloon technique was needed during treatment of LMB-ISR lesions with DCB, semi-compliant balloons were used. When two DCBs were used to treat the bifurcation lesions, they were inflated in sequential manners. First generation DES included the CYPHER® (Cordis, Johnson & Johnson, Miami Lake, Florida) and TAXUS™ (Boston Scientific Corp., Marlborough, Miami). Second generation DES included the XIENCE™ (Abbott Vascular Devices, Temecula, California) and Endeavor® series (Medtronic Cardiovascular, Santa Rosa, California). The third generation DES included the BioMatrix (Biosensors, Singapore) and Nobori (Terumo Corporation, Tokyo, Japan). In July 2010, DCB (SeQuent® Please balloon catheter, B. Braun, Melsungen, Germany) became available and was used henceforth.

### Definitions

Major adverse cardiac events (MACEs) were defined as a composite of cardiac death, non-fatal myocardial infarction (MI), target lesion revascularization, CABG, and stent thrombosis. Cardiac death was considered as a MACE, unless it was associated with a clear non-cardiac cause. MI was defined as clinically suspicious symptoms or electrocardiogram abnormalities with elevated cardiac enzymes and classified into non-ST segment elevation MI (NSTEMI) or ST segment elevation MI (STEMI). Stent thrombosis was defined as definite stent thrombosis based on the Academic Research Consortium criteria [14].

### Angiography analysis

LMB lesions were defined as the presence of narrowing of the LMCA or narrowing adjacent to (or involving) the ostium of the LAD or left circumflex artery. The distance between the ISR lesion and LAD or left circumflex artery ostium was confined to 5 mm. Bifurcation lesions were classified by two independent physicians on the basis of the Medina classification system [15]; among them, (1,1,1), (1,0,1), and (0,1,1) lesions were defined as true bifurcation lesions. ISR lesions were classified according to the Mehran classification system [16]. Multifocal, diffuse, proliferative, and occlusive ISR lesions were classified as non-focal type restenotic lesions. Stent-in-stent restenosis was defined as a second restenosis after repeat stent implantation.

Quantitative coronary angiography (QCA) analysis was performed using a quantitative coronary angiographic system (CASS system, Pie Medical Instruments, Maastricht, The Netherlands) by three radiologic technicians who were blinded to the patients’ treatment. The diameter of the reference vessels, minimal luminal diameter, and percent diameter stenosis (%) were measured from diastolic frames using guided catheter magnification-calibration in a single, matched view showing the smallest minimal luminal diameter. The acute gain was calculated as the increase in the minimal lumen diameter of the treated lesion immediately after the index procedure in contrast to that before the procedure. Late lumen loss was defined as a decrease in the minimal lumen diameter of the treated lesion at the follow-up CAG in contrast to that immediately after the index procedure. All quantitative angiographic measurements were obtained before and after PCI, and at the follow-up coronary angiography, if available.

### Statistics

Categorical variables were reported as count (percentage) and continuous variables as mean ± standard deviation. Comparisons between groups were performed using the independent Student’s t-test or Mann-Whitney test for continuous variables and the χ2 test or Fisher’s exact test for categorical variables. Kaplan-Meier survival curves with a log-rank test were generated to compare the long-term incidence of MACE between groups. Cox regression analyses were performed to compare hazard ratios during the follow-up period. The following variables were included in the Cox regression model: age, sex, current smoking status, hypertension, diabetes mellitus, prior myocardial infarction, acute MI at the index PCI, serum low density lipoprotein level, left ventricular ejection fraction, estimated glomerular filtration rate, Medina classification, ISR type, and PCI type (DES or DCB). Propensity score matching was also performed to minimize selection bias. Considering that DCB angioplasty was available after July 2010 in Korea, six patients who underwent repeat DES implantation before July 2010 were excluded for matching. We performed a 1:1 nearest neighboring matching with a default caliper distance of 0.25. Variables for matching included age, sex, current smoking status, hypertension, diabetes mellitus, acute MI at the index PCI, serum total cholesterol level, serum low-density lipoprotein cholesterol level, serum high-density lipoprotein-cholesterol level, serum triglyceride level, serum glucose level, estimated glomerular filtration rate, left ventricular ejection fraction, previous stent type, previous stent diameter and length, target vessel, stent-in-stent lesion, chronic total occlusion lesion, diffuse ISR type, true bifurcation lesion, duration between previous PCI and the index procedure, and pre-PCI angiographic measurements (target lesion reference vessel diameter, minimal lumen diameter, and lesion length). Baseline clinical and angiographic characteristics, procedural details, and QCA analyses after propensity score matching are shown in Additional file 1. The results are expressed as hazard ratio with 95% confidence interval and *p*-value. All tests were two-tailed, and *p*-values < 0.05 were considered statistically significant. All statistical analyses were performed using Statistical Package for Social Sciences software (v20, IBM SPSS Corp., Armonk, New York) and R statistical computing environment ver. 3.3.2 (R Development Core Team).

## Results

### Baseline clinical characteristics

The baseline patient characteristics included in the analysis (*n* = 75) are presented in Table 1. Baseline patient clinical characteristics in the DES (*n* = 51) and DCB (*n* = 24) groups were similar. More than 70% of patients in both groups presented with stable or unstable angina, according to the index PCI. Patients in the DES group tended to be more prone to NSTEMI/STEMI compared with those in the DCB group (25.5% vs. 8.3%; *p* = 0.12).

**Table 1:** Baseline clinical characteristics

	DES (*n* = 51)	DCB (*n* = 24)	*p*-value
Age (year)	64.3 ± 10.9	64.8 ± 11.3	0.85
Men, n (%)	41 (80.4)	17 (70.8)	0.36
Current smoker, n (%)	16 (31.4)	6 (25)	0.57
Hypertension, n (%)	34 (66.7)	14 (58.3)	0.48
Diabetes, n (%)	23 (45.1)	9 (37.5)	0.54
Prior MI, n (%)	16 (31.4)	6 (25)	0.57
Diagnosis at the index PCI			0.12
SA/UA, n (%)	38 (74.5)	22 (91.7)	
NSTEMI/STEMI, n (%)	13 (25.5)	2 (8.3)	
Laboratory findings			
Total cholesterol (mg/dL)	151.5 ± 49.3	151.8 ± 35.0	0.98
LDL-C (mg/dL)	81.7 ± 38.1	92.9 ± 35.1	0.09
HDL-C (mg/dL)	41.6 ± 12.4	41.4 ± 8.8	0.77
Triglyceride (mg/dL)	151.7 ± 83.8	134.1 ± 89.8	0.21
Glucose (mg/dL)	126.1 ± 37.3	127.4 ± 55.2	0.68
Creatinine (mg/dL)	1.13 ± 0.63	1.02 ± 0.36	0.44
hsCRP (mg/L)	7.69 ± 24.14	7.26 ± 17.98	0.87
LVEF, n (%)	52.6 ± 10.4	53.8 ± 10.7	0.41

Data were presented as n (%) or mean ± SD. DCB, drug-coated balloon; DES, drug-eluting stent; HDL-C, high density lipoprotein cholesterol; hsCRP, high sensitivity C-reactive protein; *LDL-C* low density lipoprotein cholesterol; *LVEF* left ventricular ejection fraction; *MI* myocardial infarction; *NSTEMI* non-ST segment elevation myocardial infarction; *PCI* percutaneous coronary intervention; *SA* stable angina; *UA* unstable angina; *STEMI* ST segment elevation myocardial infarction

### Lesional characteristics and procedures

Previous PCI characteristics showed that > 80% of patients in the DES group underwent 1st or 2nd generation DES implantation (Table 2). Only one patient with ISR after bare metal stent implantation was included in the DES group. In addition, the DCB group tended to have more stent-in-stent cases (25% vs. 7.8%; *p* = 0.07) and larger previous stent diameter (2.99 ± 0.29 mm vs. 2.89 ± 0.26 mm; *p* = 0.16) compared with patients in the DES group; however, the differences were not statistically significant. Among patients with LMB-ISR lesions, 33.3% (*n* = 25) had coronary arterial lesions other than LMB (e.g. proximal part of the LAD coronary artery) at the previous PCI. Lesion characteristics showed similar ISR and bifurcation patterns between groups. Approximately 50% of patients in each group had a focal type ISR. In addition, true bifurcation lesions were observed in 27.5 and 29.2% of patients in the DES and DCB groups, respectively. Second generation DES was used in approximately 70% of patients in the DES group. Procedures at the side branch, including the two-stent technique, pre-procedural or post-procedural side branch ballooning, and final kissing ballooning, were performed in 19.6 and 25% of patients in the DES and DCB groups, respectively. No cases of bail-out stenting were observed in the DCB group. QCA data also showed that the DCB group had smaller post-procedural minimal luminal diameters of the target lesion than the DES group (2.71 ± 0.29 mm vs. 2.85 ± 0.55 mm; *p* = 0.03; Table 3).

**Table 2:** Angiographic features and procedural details

	DES (*n* = 51)	DCB (*n* = 24)	*p*-value
Previous PCI characteristics			
Target lesion involving LMB, n (%)	32 (62.7)	18 (75)	0.29
Stent type			0.92
BMS, n (%)	1 (2)	0	
1st generation DES, n (%)	22 (44.9)	10 (43.5)	
2nd generation DES, n (%)	20 (40.8)	9 (39.1)	
3rd generation DES, n (%)	6 (12.2)	4 (17.4)	
Stent diameter (mm)	2.89 ± 0.26	2.99 ± 0.29	0.16
Stent length (mm)	20.50 ± 5.96	20.52 ± 7.48	0.71
Stent-in-stent, n (%)	4 (7.8)	6 (25)	0.07
Median duration between previous PCI to the index procedure (day)	1232	895	0.89
Lesion characteristics at the index PCI			
ISR pattern			0.55
Focal, n (%)	26 (51)	14 (58.3)	
Non-focal, n (%)	25 (49)	10 (41.7)	
Medina classification			0.74
0,0,1, n (%)	4 (7.8)	3 (12.5)	
0,1,0, n (%)	32 (62.7)	13 (54.2)	
0,1,1, n (%)	6 (11.8)	3 (12.5)	
1,0,0, n (%)	1 (2)	0	
1,1,0, n (%)	0	1 (4.2)	
1,1,1, n (%)	8 (15.7)	4 (16.7)	
True bifurcation, n (%)	14 (27.5)	7 (29.2)	0.88
Bifurcation angle > 90°, n (%)	25 (49)	15 (62.5)	0.28
Calcified lesion, n (%)	3 (5.9)	3 (12.5)	0.38
Chronic total occlusion, n (%)	5 (9.8)	1 (4.2)	0.66
Procedures of the index PCI			
DES type		–	–
1st generation DES, n (%)	3 (5.9)		
c2^nd^ generation DES, n (%)	35 (68.6)		
3rd generation DES, n (%)	13 (25.5)		
DES diameter (mm)	3.08 ± 0.41		
DES length (mm)	20.75 ± 8.65	–	–
Cross-over, n (%)	21 (41.2)	–	–
2-stent technique, n (%)	6 (11.8)	–	–
DCB diameter (mm)	–	3.03 ± 0.37	–
DCB length (mm)	–	19.04 ± 4.87	–
SB ballooning, n (%)	10 (19.6)	6 (25)	0.60
FK ballooning, n (%)	6 (11.8)	2 (8.3)	1.00
Intravascular imaging, n (%)	18 (35.3)	8 (33.3)	0.87

Data were presented as n (%) or mean ± SD. *BMS* bare metal stent; *DCB* drug-coated balloon; *DES* drug-eluting stent; *FK* final kissing; *ISR* in-stent restenosis; *LMB* left main bifurcation; *PCI* percutaneous coronary intervention; *SB* side branch

**Table 3:** Quantitative coronary angiography analysis

	DES	DCB	*p*-value
Pre-procedure			
n	51	24	
LMCA RVD (mm)	3.93 ± 0.60	3.86 ± 0.79	0.67
LMCA MLD (mm)	3.40 ± 1.13	3.47 ± 0.92	0.79
LMCA DS (%)	14.54 ± 23.76	10.35 ± 14.76	0.60
LAD RVD (mm)	2.72 ± 0.86	3.10 ± 0.78	0.43
LAD MLD (mm)	0.99 ± 0.84	0.98 ± 0.97	0.95
LAD DS (%)	66.57 ± 27.50	69.12 ± 27.95	0.71
LCX RVD (mm)	2.80 ± 0.74	2.91 ± 0.48	0.96
LCX MLD (mm)	1.93 ± 1.09	1.88 ± 1.23	0.94
LCX DS (%)	33.82 ± 35.98	37.00 ± 37.69	0.83
Target lesion RVD (mm)	2.94 ± 0.36	3.03 ± 0.77	0.48
Target lesion MLD (mm)	0.74 ± 0.61	0.62 ± 0.47	0.41
Target lesion DS (mm)	74.98 ± 20.09	79.75 ± 13.39	0.29
Target lesion length (mm)	19.12 ± 8.14	18.46 ± 4.56	0.59
Post-procedure			
LMCA RVD (mm)	3.96 ± 0.58	3.88 ± 0.77	0.64
LMCA MLD (mm)	3.75 ± 0.69	3.65 ± 0.66	0.58
LMCA DS (%)	5.51 ± 7.89	5.63 ± 4.63	0.94
LAD RVD (mm)	3.12 ± 0.36	3.14 ± 0.75	0.35
LAD MLD (mm)	2.81 ± 0.51	2.79 ± 0.37	0.23
LAD DS (%)	9.65 ± 13.40	9.65 ± 9.99	0.40
LCX RVD (mm)	2.97 ± 0.45	2.92 ± 0.49	0.55
LCX MLD (mm)	2.51 ± 0.63	2.59 ± 0.67	0.60
LCX DS (%)	15.45 ± 18.54	11.99 ± 15.07	0.33
Target lesion RVD (mm)	3.12 ± 0.39	3.08 ± 0.74	0.11
Target lesion MLD (mm)	2.85 ± 0.55	2.71 ± 0.29	0.03
Target lesion DS (%)	8.68 ± 12.70	10.00 ± 9.91	0.12
Acute gain (mm)	2.12 ± 0.80	2.09 ± 0.50	0.89
Follow-up			
n	25	13	
Median follow-up period (day)	294	560	0.66
LMCA RVD (mm)	3.91 ± 0.55	3.70 ± 0.56	0.27
LMCA MLD (mm)	3.42 ± 0.94	3.25 ± 0.75	0.59
LMCA DS (%)	12.76 ± 19.60	12.51 ± 11.68	0.57
LAD RVD (mm)	3.07 ± 0.67	3.04 ± 0.44	0.87
LAD MLD (mm)	2.33 ± 1.01	2.01 ± 1.09	0.37
LAD DS (%)	20.01 ± 48.10	33.07 ± 33.83	0.85
LCX RVD (mm)	2.76 ± 0.40	2.71 ± 0.74	0.69
LCX MLD (mm)	1.79 ± 1.00	1.61 ± 0.91	0.37
LCX DS (%)	35.85 ± 35.67	40.70 ± 30.60	0.59
Target lesion RVD (mm)	3.00 ± 0.71	2.91 ± 0.35	0.53
Target lesion MLD (mm)	2.34 ± 1.04	1.68 ± 0.96	0.04
Target lesion DS (%)	19.61 ± 48.63	40.37 ± 34.61	0.31
Late lumen loss (mm)	0.60 ± 0.85	1.06 ± 1.10	0.23
Binary restenosis, n (%)	5 (20)	6 (46.2)	0.14

Data were presented as n (%) or mean ± SD. *DCB* drug-coated balloon; *DES* drug-eluting stent; *DS* diameter stenosis; *LAD* left anterior descending artery; *LCX* left circumflex artery; *LMCA* left main coronary artery; *MLD* minimal lumen diameter; *RVD* reference vessel diameter

### Follow-up coronary angiography

Among the patients, 47% (25/51) and 54% (13/24) of patients in the DES and DCB groups, respectively, underwent follow-up CAG. QCA data of the follow-up CAG also showed that patients in the DCB group had smaller minimal luminal diameters of the target lesion (1.68 ± 0.96 mm vs. 2.34 ± 1.04 mm; *p* = 0.04), larger late lumen loss (1.06 ± 1.10 mm vs. 0.60 ± 0.85 mm; *p* = 0.23), and higher binary restenosis rates (46.2% vs. 20%; *p* = 0.14) than patients in the DES group.

### Clinical outcome

**Fig. 1:**
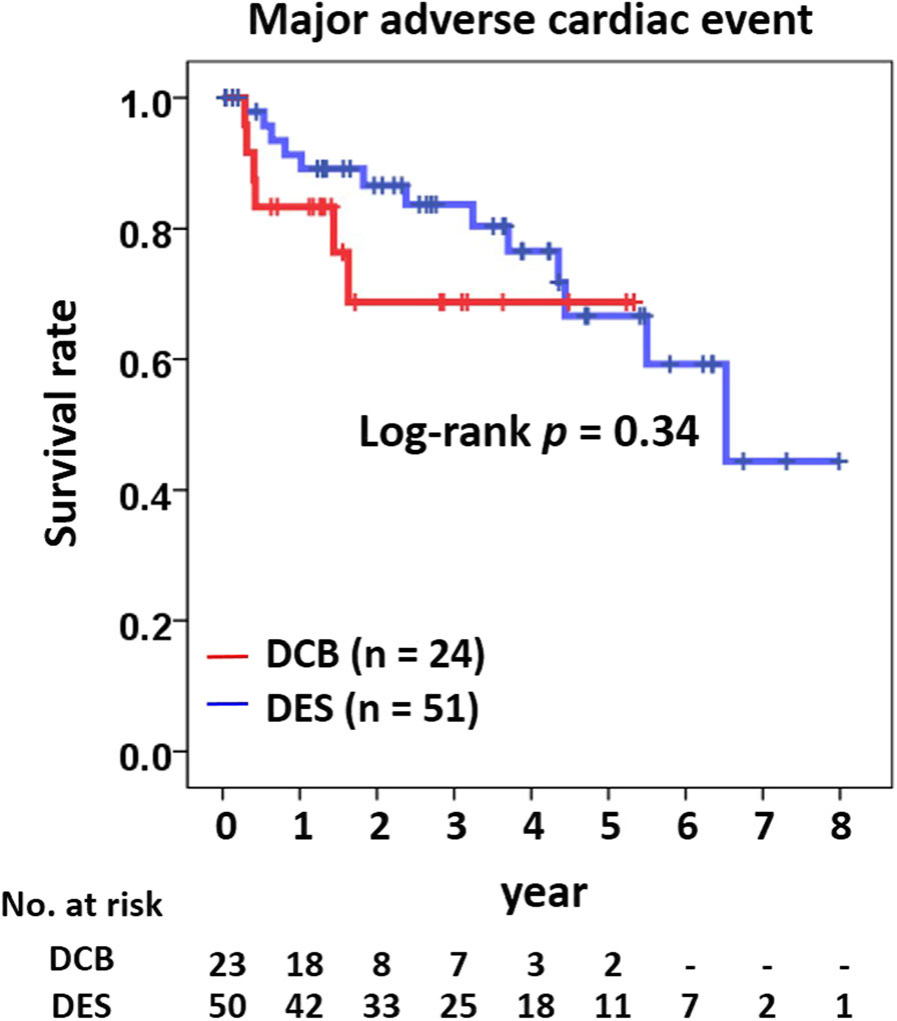
Kaplan-Meier curve for major adverse cardiac events. DCB, drug-coated stent; DES, drug-eluting stent

The median follow-up duration for all patients (*n* = 75) was 868 days with 1009 days and 520 days in the DES and DCB groups, respectively (*p* = 0.02). The cumulative incidence rate of MACE was similar between the DES and DCB groups (25.5 and 25%, respectively; *p* = 0.96; Table 4). There was one cardiac death, one non-fatal MI, and two stent thrombosis cases in the DES group. Target lesion revascularization occurred in 11 (21.6%) and 4 (16.7%) patients in the DES and DCB groups, respectively (*p* = 0.76). CABG was performed in two (3.9%) and three (12.5%) patients in the DES and DCB groups, respectively (*p* = 0.32). No significant differences were observed for the 1-year MACE rates between the DES and DCB groups (17.6 and 25%, respectively; *p* = 0.54). Kaplan-Meier analysis demonstrated that the long-term incidence of MACEs was similar between the groups (log-rank test, *p* = 0.34; Fig. 1). In the propensity score matching analysis (24 patients in each group), the cumulative incidence rates of MACE were also similar between the groups (25 and 29.2% in the DCB and DES groups, respectively; log-rank test, *p* = 0.64; Additional file 2). Multivariate Cox regression analysis demonstrated that only true bifurcation ISR lesions were an independent predictor for MACE (Table 5).

**Table 4:** The cumulative incidence of clinical events

	DES (*n* = 51)	DCB (*n* = 24)	*p*-value
Major adverse cardiac event, *n* (%)	13 (25.5)	6 (25)	0.96
All-cause death, *n* (%)	2 (3.9)	0	1.00
Cardiac death, n (%)	1 (2)	0	1.00
Non-fatal myocardial infarction, *n* (%)	1 (2)	0	1.00
Target lesion revascularization, *n* (%)	11 (21.6)	4 (16.7)	0.76
Coronary artery bypass graft, *n* (%)	2 (3.9)	3 (12.5)	0.32
Stent thrombosis, *n* (%)	2 (3.9)	0	1.00

Data were presented as n (%) or mean ± SD. *DCB* drug-coated balloon; *DES* drug-eluting stent

**Table 5:** Cox-proportional hazard models for major adverse cardiac event

	HR	95% CI	*p*-value
Univariate			
Age > 65 years	1.29	0.517–3.237	0.58
Men	1.21	0.434–3.367	0.72
Current smoking	1.55	0.596–4.012	0.37
Hypertension	4.04	0.931–17.527	0.06
Diabetes mellitus	0.53	0.201–1.400	0.20
Prior MI history	1.94	0.749–5.036	0.17
NSTEMI/STEMI at index PCI	0.89	0.290–2.736	0.84
LDL-C > 70 mg/dL	0.88	0.333–2.329	0.80
eGFR < 60 ml/min/1.73m^2^	1.05	0.348–3.175	0.93
LVEF < 40%	1.85	0.412–8.255	0.42
Previous LMB lesion	1.89	0.620–5.735	0.26
Previous 1st generation DES	1.46	0.582–3.657	0.42
Stent-in-stent	0.53	0.122–2.335	0.40
True bifurcation	3.88	1.545–9.743	< 0.01
ISR type (III, IV)	1.16	0.471–2.871	0.74
DCB (vs. DES)	1.64	0.595–4.509	0.34
Multivariate			
True bifurcation	4.53	1.476–13.898	< 0.01

*95% CI* 95% confidence interval; *DCB* drug-coated balloon; *DES* drug-eluting stent; *eGFR* estimated glomerular filtration rate; *HR* hazard ratio; *ISR* in-stent restenosis; *LDL-C* low density lipoprotein cholesterol; *LVEF* left ventricular ejection fraction; *NSTEMI* non-ST segment elevation myocardial infarction; *STEMI* ST segment elevation myocardial infarction

## Discussion

To our knowledge, this is the first report comparing the long-term clinical outcomes of DES and DCB in patients with in-stent restenotic lesions involving LMB. Initially, 33.3% of patients with LMB-ISR lesions had no prior LMB lesions during the previous PCI. In addition, the incidence of MACE was similar between the groups during the median follow-up period (868 days) according to Kaplan–Meier and multivariate Cox regression analyses. Further, true bifurcation lesions were found to be an important independent predictor for MACE after the procedure.

Previous clinical studies showed that the majority (65–95%) of patients with LMCA ISR presented with stable angina or were asymptomatic [8, 9]. However, the treatment of choice for those patients was usually repeat PCI, because differentiating benign non-functional angiographic restenosis from obstructive ischemia-inducing functional restenosis is more difficult in LMB-ISR lesions in contrast to any other coronary vasculature [17]. Among the various PCI options for patients with ISR lesions—including balloon angioplasty, vascular brachytherapy, or rotablation—DCB angioplasty and repeat DES implantation have shown more favorable angiographic and clinical outcomes [18]. Recent ESC guidelines recommend DCB angioplasty and repeat DES implantation as class IA [4]. The uncertainty regarding the clinical prognosis between DCB angioplasty and repeat DES implantation may reflect the importance of clinical judgement in choosing the optimal treatment option in each case. Although the present study failed to show the statistical difference in baseline clinical, angiographic, and procedural details, there were several trends differentiating the groups. In particular, patients presenting with NSTEMI/STEMI favored DES implantation in contrast to DCB (8.3% vs. 25.5%, *p* = 0.12). The DCB group tended to have a larger previous stent diameter (2.99 ± 0.29 mm vs. 2.89 ± 0.26 mm, *p* = 0.16) and more frequent stent-in-stent cases (25% vs. 7.8%; *p* = 0.07) than the DES group.

There are several randomized trials comparing the efficacy between repeat DES implantation and DCB angioplasty in patients with DES-ISR lesions. The ISAR-DESIRE 3 and PEPCAD China ISR trials showed non-inferior angiographic and clinical outcomes in patients who underwent DCB angioplasty when compared with 1st generation paclitaxel-eluting stent implantation [13, 19, 20]. In contrast, the RIBS IV trials showed that 2nd generation everolimus-eluting stent implantation had superior angiographic and clinical results when compared with DCB angioplasty [21]. A recent network meta-analysis suggested using DES implantation and DCB angioplasty in patients with ISR lesions and 2nd generation everolimus-eluting stents over any other treatment modality [22].. In the present study, 2nd and 3rd generation DES were used in 94.1% of patients in the DES group. Further, the current study demonstrated similar trends favoring DES implantation for better angiographic outcomes when compared with DCB angioplasty although the MACE rates were comparable between the groups.

Nevertheless, there have been several concerns that the multiple metal layers in the coronary arteries left behind by repeat DES implantation could contribute to the increased risk of recurrent restenosis or stent thrombosis owing to the potential hazard of delayed re-endothelization and inflammation. In a network meta-analysis, Lee et al. reported that DES implantation had a trend towards a higher MI risk when compared with DCB angioplasty [23]. Furthermore, additional side branch interventions, such as the kissing balloon technique, may induce the deformation of implanted stent struts in patients with bifurcation ISR lesions. A retrospective analysis of 683 patient with DES-ISR lesions showed a potential benefit of DCB angioplasty in non-focal type and bifurcation lesions in contrast to repeat DES implantation [24]. However, Naganuma et al. reported similar MACE rates between DCB angioplasty and DES implantation in 158 patients with bifurcation ISR lesions [25]. Although our study showed similar clinical results between DCB angioplasty and DES implantation, closer attention should be paid to the higher number of cardiac death, myocardial infarction, and stent thrombosis cases we observed in the DES group.

True bifurcation lesions remain the Achilles’ heel of PCI. The TRYTON trial showed high failure rates of target vessel at the 9-month follow-up (12–18%), even in de novo true bifurcation lesions [26]. The cumulative incidence of MACE was 47.6% in patients with true LMB-ISR lesions in contrast to 16.7% in those without (*p* < 0.01). Multivariate Cox-regression analysis also showed that true bifurcation ISR lesions were the only independent prognostic factor for poor MACE-related outcomes (hazard ratio, 4.53; 95% confidence interval, 1.476–13.898; *p* < 0.01). Therefore, other treatment strategies including CABG should be considered for such patients.

There are several limitations of our study. First, we only included angiographic assessment data due to the limited usage (34.7%) of additional intravascular imaging tools, including intravascular ultrasound or optical coherence tomography. Angiographic assessments could have limited the detailed lesion and procedural information available for this study. Moreover, only half of the patients underwent follow-up angiography, which also undermined the statistical significance. Changes in the treated vessels assessed with follow-up angiography may better reflect the effect of each treatment modality rather than MACE. However, due to the inherent limitations of the study design, we were unable to obtain follow-up angiographic data in all study populations. Future prospective studies designed to assess the coronary angiographic changes before and after treatment of LMB-ISR lesions in these particular populations may overcome the limitations of the current study. Second, this was a retrospective observational study. As the treatment strategy was decided at the discretion of the surgeons, selection bias was inevitable and may have limited our interpretation. For example, DCB was more frequently used for restenosis of a double-stent strut layer rather than that of a single-stent strut layer of the previous stent. Moreover, the operator’s experience or personal preference for DCB may have also affected the treatment strategy selected. Third, the sample size was too small to perform powerful comparative statistical analyses between DES implantation and DCB angioplasty. However, the study population was enrolled for a long duration, although DCB only became available in July 2010. The clinical, lesional, and procedural characteristics of the patients enrolled were also highly heterogeneous. Thus, our findings may not be generalizable to all LMB-ISR treatments. Studies with a large number of participants are needed to validate these results. Despite these limitations, a significant strength of the present study is that it is the first study comparing DES implantation to DCB angioplasty in patients with LMB-ISR lesions.

## Conclusion

Both DCB angioplasty and repeat DES implantation strategies for LMB-ISR lesion showed similar clinical outcomes in terms of MACE in the long-term follow-up.

## Supplementary information

**Additional file 1:Supplemental table 1.** Baseline clinical and angiographic characteristics and procedural details after the propensity score matching. **Supplemental table 2.** Quantitative coronary angiography analysis after the propensity score matching.

**Additional file 2:** A. The cumulative incidence of clinical events after propensity score matching. B. Kaplan-Meier curve for major adverse cardiac events after propensity score matching.

## Data Availability

Raw data supporting the obtained results are available with the corresponding author.

## Abbreviations

CABG: Coronary artery bypass graft surgery
DCB: Drug-coated balloon
DES: Drug-eluting stent
ESC: European Society of Cardiology
ISR: In-stent restenosis
LAD: Left anterior descending artery
LMB: Left main bifurcation
LMCA: Left main coronary artery
MACE: Major adverse cardiac event
MI: Myocardial infarction
NSTEMI: Non-ST segment elevation myocardial infarction
PCI: Percutaneous coronary intervention
QCA: Quantitative coronary angiography
STEMI: ST segment elevation myocardial infarction
