# Supplementary material for: A comparison between drug-eluting stent implantation and drug-coated balloon angioplasty in patients with left main bifurcation in-stent restenotic lesions
Source: BMC Cardiovasc Disord. 2020 Feb 18;20:83. doi: 10.1186/s12872-020-01381-9 (PMC7027103; doi:10.1186/s12872-020-01381-9)
Supplement: Supplementary file 1 — Additional file 1:Supplemental table 1. Baseline clinical and angiographic characteristics and procedural details after the propensity score matching. Supplemental table 2. Quantitative coronary angiography analysis after the propensity score matching. [file 12872_2020_1381_MOESM1_ESM.docx]

**SUPPLEMENTAL TABLE**

**Supplemental table 1.** Baseline clinical and angiographic characteristics and procedural details after the propensity score matching

|  | DES  (n=24) | DCB  (n=24) | *p*-value |
| --- | --- | --- | --- |
| Age (year) | 67.0 ± 11.0 | 64.8 ± 11.3 | 0.49 |
| Men, n (%) | 18 (75) | 17 (70.8) | 0.75 |
| Current smoker, n (%) | 7 (29.2) | 6 (25) | 0.75 |
| Hypertension, n (%) | 16 (66.7) | 14 (58.3) | 0.55 |
| Diabetes, n (%) | 10 (41.7) | 9 (37.5) | 0.77 |
| Prior MI, n (%) | 8 (33.3) | 6 (25) | 0.53 |
| Diagnosis at the index PCI  SA/UA, n (%)  NSTEMI/STEMI, n (%) | 18 (75)  6 (25) | 22 (91.7)  2 (8.3) | 0.25 |
| **Laboratory findings** | | | |
| Total cholesterol (mg/dL) | 145.1 ± 3.51 | 149.7 ± 35.9 | 0.66 |
| LDL-C (mg/dL) | 78.0± 30.7 | 92.9 ± 35.1 | 0.07 |
| HDL-C (mg/dL) | 45.1 ± 12.6 | 41.4 ± 8.8 | 0.34 |
| Triglyceride (mg/dL) | 132.3 ± 65.8 | 134.1 ± 89.8 | 0.60 |
| Glucose (mg/dL) | 120.2 ± 28.3 | 127.4 ± 55.2 | 0.95 |
| Creatinine (mg/dL) | 1.16 ± 0.69 | 1.02 ± 0.36 | 0.46 |
| LVEF, n (%) | 52.4 ± 10.7 | 53.8 ± 10.7 | 0.45 |
| **Previous PCI characteristics** | | | |
| Target lesion involving LMB, n (%) | 15 (62.5) | 18 (75) | 0.35 |
| Stent type  BMS, n (%)  1^st^ generation DES, n (%)  2^nd^ generation DES, n (%)  3^rd^ generation DES, n (%) | 1 (4.2)  12 (50)  9 (37.5)  2 (8.3) | 0 (0)  10 (41.7)  10 (41.7)  4 (16.7) | 0.72 |
| Stent diameter (mm) | 2.86 ± 0.30 | 3.01 ± 0.30 | 0.06 |
| Stent length (mm) | 21.0 ± 6.5 | 20.4 ± 7.4 | 0.54 |
| Stent-in-stent, n (%) | 1 (4.2) | 6 ( 25) | 0.10 |
| Median duration between previous PCI to the index procedure (day) | 1582 ± 976 (1655) | 1459 ± 1220 (895.5) | 0.52 |
| **Lesion characteristics at the index PCI** | | | |
| ISR pattern  Focal, n (%)  Non-focal, n (%) | 13 (54.2)  11 (45.8) | 14 (58.3)  10 (41.7) | 0.77 |
| Medina classification  0,0,1, n (%)  0,1,0, n (%)  0,1,1, n (%)  1,0,0, n (%)  1,1,0, n (%)  1,1,1, n (%) | 4 (16.7)  12 (50)  2 (8.3)  0 (0)  0 (0)  6 (25) | 3 (12.5)  13 (54.2)  3 (12.5)  0 (0)  1 (4.2)  4 (16.7) | 0.89 |
| True bifurcation, n (%) | 8 (33.3) | 7 (29.2) | 0.76 |
| Bifurcation angle > 90°, n (%) | 13 (54.2) | 15 (62.5) | 0.56 |
| Calcified lesion, n (%) | 1 (4.2) | 3 (12.5) | 0.61 |
| Chronic total occlusion, n (%) | 2 (8.3) | 1 (4.2) | 1.00 |
| **Procedures of the index PCI** | | | |
| DES type  2^nd^ generation DES, n (%)  3^rd^ generation DES, n (%) | 16 (66.7)  8( 33.3) | - | - |
| DES diameter (mm) | 3.03 ± 0.41 |  |  |
| DES length (mm) | 20.54 ± 9.33 | - | - |
| Cross-over, n (%) | 13 (54.2) | - | - |
| 2-stent technique, n (%) | 4 (16.7) | - | - |
| DCB diameter (mm) | - | 3.03 ± 0.37 | - |
| DCB length (mm) | - | 19.04 ± 4.87 | - |
| SB ballooning, n (%) | 6 (25) | 6 (25) | 1.00 |
| FK ballooning, n (%) | 4 (16.7) | 2 (8.3) | 0.67 |
| Intravascular imaging, n (%) | 9 (37.5) | 8 (33.3) | 0.76 |

Data were presented as n (%) or mean ± SD. DCB, drug-coated balloon; DES, drug-eluting stent; HDL-C, high density lipoprotein cholesterol; hsCRP, high sensitivity C-reactive protein; LDL-C, low density lipoprotein cholesterol; LVEF, left ventricular ejection fraction; MI, myocardial infarction; NSTEMI, non-ST segment elevation myocardial infarction; PCI, percutaneous coronary intervention; SA, stable angina; UA, unstable angina; STEMI, ST segment elevation myocardial infarction; BMS, bare metal stent; FK, final kissing; ISR, in-stent restenosis; LMB, left main bifurcation; PCI, percutaneous coronary intervention; SB, side branch.

**Supplemental table 2.** Quantitative coronary angiography analysis after the propensity score matching

|  | DES | DCB | *p*-value |
| --- | --- | --- | --- |
| **Pre-procedure** | | | |
| n | 24 | 24 |  |
| LMCA RVD (mm) | 4.01 ± 0.57 | 3.86 ± 0.79 | 0.22 |
| LMCA MLD (mm) | 3.48 ± 1.06 | 3.47 ± 0.92 | 0.97 |
| LMCA DS (%) | 13.91 ± 21.54 | 10.35 ± 14.76 | 0.60 |
| LAD RVD (mm) | 2.87 ± 0.72 | 3.10 ± 0.78 | 0.86 |
| LAD MLD (mm) | 1.12 ± 0.92 | 0.98 ± 0.97 | 0.46 |
| LAD DS (%) | 63.06 ± 28.80 | 69.12 ± 27.95 | 0.39 |
| LCX RVD (mm) | 2.68 ± 0.96 | 2.91 ± 0.48 | 0.89 |
| LCX MLD (mm) | 1.73 ± 1.25 | 1.88 ± 1.23 | 0.68 |
| LCX DS (%) | 42.29 ± 38.70 | 37.00 ± 37.69 | 0.23 |
| Target lesion RVD (mm) | 2.93 ± 0.40 | 3.03 ± 0.77 | 0.93 |
| Target lesion MLD (mm) | 0.71 ± 0.64 | 0.62 ± 0.47 | 0.75 |
| Target lesion DS (%) | 75.67 ± 21.25 | 79.75 ± 13.39 | 0.43 |
| Target lesion length (mm) | 18.96 ± 8.47 | 18.46 ± 4.56 | 0.54 |
| **Post-procedure** | | | |
| LMCA RVD (mm) | 4.04 ± 0.58 | 3.88 ± 0.77 | 0.43 |
| LMCA MLD (mm) | 3.87 ± 0.61 | 3.65 ± 0.66 | 0.25 |
| LMCA DS (%) | 4.29 ± 3.77 | 5.63 ± 4.63 | 0.28 |
| LAD RVD (mm) | 3.15 ± 0.39 | 3.14 ± 0.75 | 0.36 |
| LAD MLD (mm) | 2.74 ± 0.64 | 2.79 ± 0.37 | 0.73 |
| LAD DS (%) | 12.87 ± 17.99 | 9.65 ± 9.99 | 0.97 |
| LCX RVD (mm) | 2.98 ± 0.45 | 2.92 ± 0.49 | 0.67 |
| LCX MLD (mm) | 2.41 ± 0.81 | 2.59 ± 0.67 | 0.65 |
| LCX DS (%) | 20.13 ± 22.40 | 11.99 ± 15.07 | 0.11 |
| Target lesion RVD (mm) | 3.11 ± 0.41 | 3.08 ± 0.74 | 0.20 |
| Target lesion MLD (mm) | 2.77 ± 0.68 | 2.71 ± 0.29 | 0.67 |
| Target lesion DS (%) | 11.18 ± 17.07 | 10.00 ± 9.91 | 0.56 |
| Acute gain (mm) | 2.07 ± 0.89 | 2.09 ± 0.50 | 0.91 |
| **Follow-up** | | | |
| n | 14 | 13 |  |
| Median follow-up period (day) | 620 | 560 | 0.40 |
| LMCA RVD (mm) | 4.08 ± 0.59 | 3.70 ± 0.56 | 0.09 |
| LMCA MLD (mm) | 3.55 ± 0.99 | 3.25 ± 0.75 | 0.39 |
| LMCA DS (%) | 13.50 ± 19.15 | 12.51 ± 11.68 | 0.87 |
| LAD RVD (mm) | 3.12 ± 0.38 | 3.04 ± 0.44 | 0.63 |
| LAD MLD (mm) | 2.06 ± 1.17 | 2.01 ± 1.09 | 0.92 |
| LAD DS (%) | 35.34 ± 35.16 | 33.07 ± 33.83 | 0.87 |
| LCX RVD (mm) | 2.76 ± 0.42 | 2.71 ± 0.74 | 0.40 |
| LCX MLD (mm) | 1.49 ± 1.19 | 1.61 ± 0.91 | 0.77 |
| LCX DS (%) | 48.87 ± 40.03 | 40.70 ± 30.60 | 0.56 |
| Target lesion RVD (mm) | 2.99 ± 0.52 | 2.91 ± 0.35 | 0.66 |
| Target lesion MLD (mm) | 2.06 ± 1.21 | 1.68 ± 0.96 | 0.37 |
| Target lesion DS (%) | 34.58 ± 36.89 | 40.37 ± 34.61 | 0.68 |
| Late lumen loss (mm) | 0.84 ± 1.15 | 1.06 ± 1.10 | 0.62 |
| Binary restenosis, n (%) | 4 (28.6) | 6 (46.2) | 0.44 |

Data were presented as n (%) or mean ± SD. DCB, drug-coated balloon; DES, drug-eluting stent; DS, diameter stenosis; LAD, left anterior descending artery; LCX, left circumflex artery; LMCA, left main coronary artery; MLD, minimal lumen diameter; RVD, reference vessel diameter.
