# Supplementary material for: A comparison between drug-eluting stent implantation and drug-coated balloon angioplasty in patients with left main bifurcation in-stent restenotic lesions
Source: BMC Cardiovasc Disord. 2020 Feb 18;20:83. doi: 10.1186/s12872-020-01381-9 (PMC7027103; doi:10.1186/s12872-020-01381-9)
Supplement: Supplementary file 2 — Additional file 2: A. The cumulative incidence of clinical events after propensity score matching. B. Kaplan-Meier curve for major adverse cardiac events after propensity score matching. [file 12872_2020_1381_MOESM2_ESM.pptx]

## Slide 1
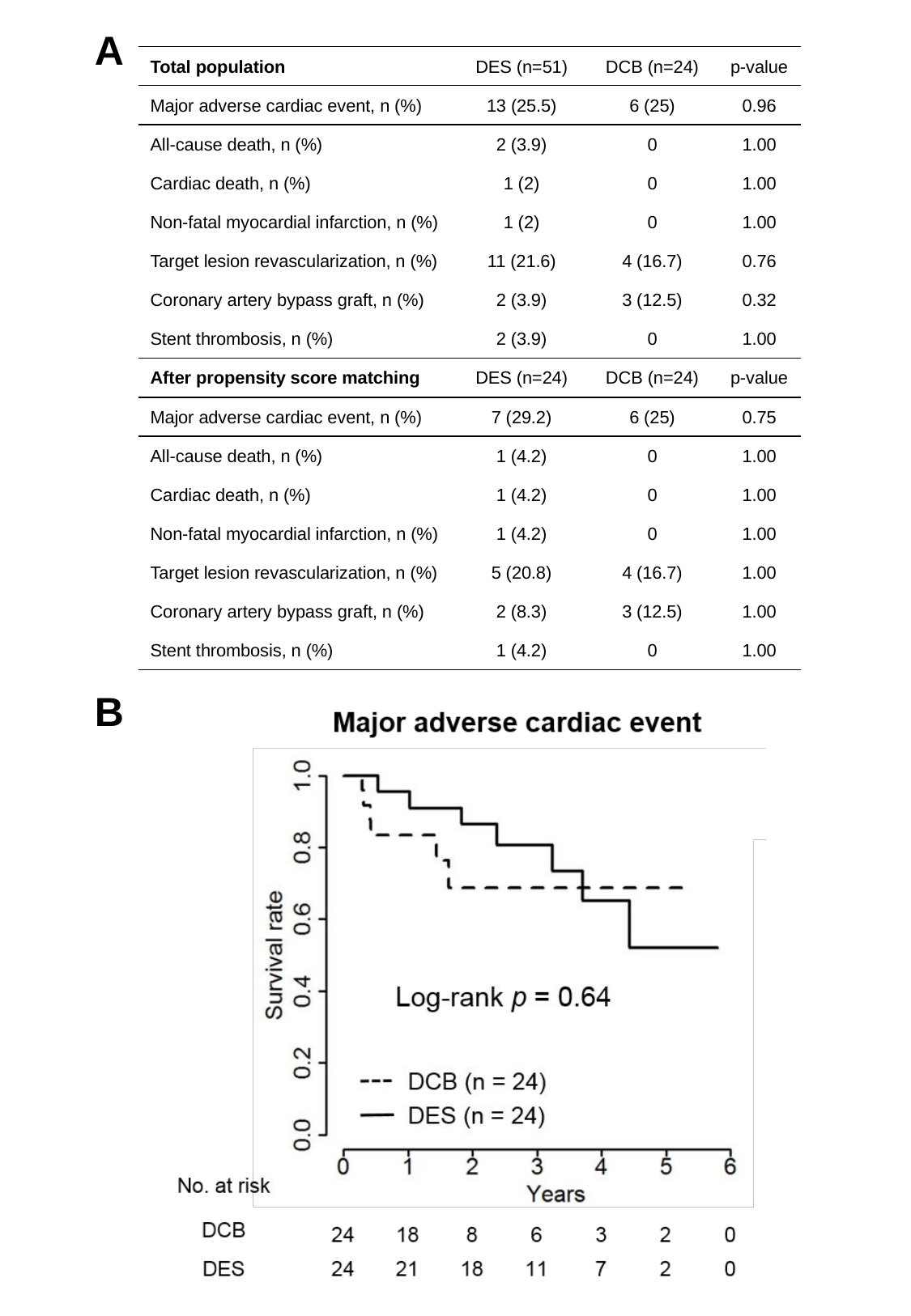

A
| Total population | DES (n=51) | DCB (n=24) | p-value |
| --- | --- | --- | --- |
| Major adverse cardiac event, n (%) | 13 (25.5) | 6 (25) | 0.96 |
| All-cause death, n (%) | 2 (3.9) | 0 | 1.00 |
| Cardiac death, n (%) | 1 (2) | 0 | 1.00 |
| Non-fatal myocardial infarction, n (%) | 1 (2) | 0 | 1.00 |
| Target lesion revascularization, n (%) | 11 (21.6) | 4 (16.7) | 0.76 |
| Coronary artery bypass graft, n (%) | 2 (3.9) | 3 (12.5) | 0.32 |
| Stent thrombosis, n (%) | 2 (3.9) | 0 | 1.00 |
| After propensity score matching | DES (n=24) | DCB (n=24) | p-value |
| Major adverse cardiac event, n (%) | 7 (29.2) | 6 (25) | 0.75 |
| All-cause death, n (%) | 1 (4.2) | 0 | 1.00 |
| Cardiac death, n (%) | 1 (4.2) | 0 | 1.00 |
| Non-fatal myocardial infarction, n (%) | 1 (4.2) | 0 | 1.00 |
| Target lesion revascularization, n (%) | 5 (20.8) | 4 (16.7) | 1.00 |
| Coronary artery bypass graft, n (%) | 2 (8.3) | 3 (12.5) | 1.00 |
| Stent thrombosis, n (%) | 1 (4.2) | 0 | 1.00 |
B
